# Knowledge, attitude and practice of healthcare ethics among resident doctors and ward nurses from a resource poor setting, Nepal

**DOI:** 10.1186/s12910-016-0154-9

**Published:** 2016-11-08

**Authors:** Samaj Adhikari, Kumar Paudel, Arja R. Aro, Tara Ballav Adhikari, Bipin Adhikari, Shiva Raj Mishra

**Affiliations:** 1 Institute of Medicine, Maharajgunj Medical Campus, Tribhuvan University, Kathmandu, Nepal; 2 Institute of Medicine, Maharajgunj Medical Campus, Tribhuvan University, Kathmandu, Nepal; 3 Unit for Health Promotion Research, Institute of Public Health, University of Southern Denmark, Niels Bohrsvej 9, Esbjerg, 6700 Denmark; 4 Faculty of Health Sciences, Unit for Health Promotion Research, University of Southern Denmark, Esbjerg, Denmark; 5 Mahidol-Oxford Tropical Medicine Research Unit, Faculty of Tropical Medicine, Mahidol University, Bangkok, Thailand; 6 Nepal Development Society, Bharatpur, 10 Chitwan Nepal

**Keywords:** Medical ethics, Cross-sectional studies, Medical doctor, Nurse, Nepal

## Abstract

**Background:**

Healthcare ethics is neglected in clinical practice in LMICs (Low and Middle Income Countries) such as Nepal. The main objective of this study was to assess the current status of knowledge, attitude and practice of healthcare ethics among resident doctors and ward nurses in a tertiary teaching hospital in Nepal.

**Methods:**

This was a cross sectional study conducted among resident doctors (*n* = 118) and ward nurses (*n* = 86) in the largest tertiary care teaching hospital of Nepal during January- February 2016 with a self-administered questionnaire. A Cramer’s V value was assessed to ascertain the strength of the differences in the variables between doctors and nurses. Association of variables were determined by Chi square and statistical significance was considered if p value was less than 0.05.

**Results:**

Our study demonstrated that a significant proportion of the doctors and nurses were unaware of major documents of healthcare ethics: Hippocratic Oath (33 % of doctors and 51 % of nurses were unaware), Nuremberg code (90 % of both groups were unaware) and Helsinki Declaration (85 % of doctors and 88 % of nurses were unaware). A high percentage of respondents said that their major source of information on healthcare ethics were lectures (67.5 % doctors versus 56.6 % nurses), books (62.4 % doctors versus 89.2 % nurses), and journals (59 % doctors versus 89.2 % nurses). Attitude of doctors and nurses were significantly different (*p* < 0.05) in 9 out of 22 questions pertaining to different aspects of healthcare ethics. More nurses had agreement than doctors on the tested statements pertaining to different aspects of healthcare ethics except for need of integration of medical ethics in ungraduate curricula (97.4 % doctors versus 81.3 % nurses),paternalistic attitude of doctor was disagreed more by doctors (20.3 % doctors versus 9.3 % nurses). Notably, only few (9.3 % doctors versus 14.0 % nurses) doctors stood in support of physician-assisted dying.

**Conclusions:**

Significant proportion of doctors and nurses were unaware of three major documents on healthcare ethics which are the core principles in clinical practice. Provided that a high percentage of respondents had motivation for learning medical ethics and asked for inclusion of medical ethics in the curriculum, it is imperative to avail information on medical ethics through subscription of journals and books on ethics in medical libraries in addition to lectures and training at workplace on medical ethics which can significantly improve the current paucity of knowledge on medical ethics.

**Electronic supplementary material:**

The online version of this article (doi:10.1186/s12910-016-0154-9) contains supplementary material, which is available to authorized users.

## Background

The Hippocratic Oath which forms the moral ground of clinical practice, is currently viewed dialectically. With inexorable progress in medicine and commercialization, the classical basis of ethical aspects of clinical practice is redefined in following major documents like Nuremberg code and Helsinki declaration. The relevance of healthcare ethics in a particular country parallels with prevailing law. Moreover, economic constraints and contemporary social values often shape and determine ethical practice.

Healthcare ethics is a sensitive framework embedded within the professionalism of medical personnel. Non-adherence to healthcare ethics and unsatisfactory management and solution of the cases not only threaten to impair doctor-patient relationships, but may also lead to suboptimal service delivery and potentially trigger incidences of violence and abuse. In various settings, evidence of unethical conduct observed by medical students, resident doctors and nurses have been reported [1–3]. The four basic principles of medical ethics (autonomy, justice, beneficence and non-maleficence) form the foundation for health professionals to guide and decide what practices are ethical in clinical settings [4, 5]. These basic ethical principles are grounded on the major documents of healthcare ethics (Hippocratic Oath, Nuremberg code and Helsinki declaration). Future doctors and nurses are expected to learn and abide by these ethical principles and documents as early as possible in their career. This warrants appropriate education of such principles; however, challenges remain in resource-poor settings such as Nepal, where curricula barely mandates the teaching of medical ethics didactically. In addition, teaching and drills on medical ethics during early clinical practice for medical students are often overlooked and are thus deprioritized [6]. Students and junior doctors in medical schools have been found to learn healthcare ethics subtly via the seniors popularly termed as the hidden or silent curriculum [7].

Physicians and nurses are the key pillars of healthcare delivery, however, as they differ by education, professional responsibilities and perceived medical norms and conducts, there are urgent need of standardization and uniformity in medical ethics among all health care professionals [8–10]. Lack of uniformity in health care ethics can inevitably allow doctors and nurses to practice in a way which are justified by their own perceived norms and conducts.

### Medical ethics in Nepal

In Nepal, ethical principles (Nepal Medical Council Act) and consumer protection act during health care are largely neglected. Health care practitioners and health care receivers are legally guided by Nepal Medical Council Cct 1968 [11] and consumer protection act 1998 [12]. However, lack of implementation of medical ethics and consumer protection act in health care can be attributed largely to poor governance and impunity [13, 14]. Health care in Nepal are largely jeopardized by the commercialization, lack of awareness concerning medical ethics and lack of litigation among health care providers and receivers. Nearly 80 % of health expenditure is out of pocket owing to lack of risk pooling scheme like social insurance [15]. As a result, conflicts arising due to practitioners’ negligence and natural outcome such as death during the treatment are reacted by beneficiaries through disrespect and violence (threats, bargain on financial compensation, vandalization of health care institution and psychosocial torture) [16]. While litigation as a result of awareness are increasing in a negligible proportion in recent years, the ongoing violence has already incurred huge amount of physical and psychosocial damages [14]. An urgent measures are required; such as to supervise the ethical practice, protection of health care practitioners, protection of consumers and litigation to discourse the current trend.

The widespread challenges in ethical governance of medical practices in Nepal can be traced to medical education. Nearly 1,451 staff nurses and 1,074 Bachelor of Medicine and Bachelor of Surgery (MBBS) doctors are produced annually [17]. Medical students receive a British-model MBBS degree: four and half years of theory and 1 year of internship. Curriculum in medical schools is community based, system based and integrated [18]. The curriculum has been revised in the last 6 years rigorously, nevertheless, healthcare ethics has remained neglected. In addition, based on anecdotal experience of authors (health care workers) in medical schools in Nepal, medical graduates hardly get 10 h of formal lectures and trainings on healthcare ethics during their entire course of study. Medical ethics in current curriculum relies on the department of forensic science and are largely limited to forensic cases. Similar is the case for nurses who are scarcely trained about medical ethics.

Nepal Medical Council (NMC) recommends the model curriculum and study materials for teaching healthcare ethics prepared by WHO Regional Office for South-East Asia [19]. However, imparting knowledge on healthcare ethics is limited to few lecture hours. Within these few hours students are expected to learn major codes of medical ethics, malpractice, negligence, consent and the duties and rights of practitioners.

NMC is the responsible body for the standardization of medical education and medical practice which ensures the implementation of Code of Ethics on accordance with Nepal Medical Council Act 1968. The Code of Ethics prevents perceived professional misconducts such as the abuse of professional privileges, defying on professional duties and breach on medical ethics which are considered as professional misconducts. This Code has laid out clearly that these misconducts can lead to dismissal of the medical licence and permanent retraction of the name of practitioner from the NMC’s register [11]. However, this act has remained largely unimplemented due to several ethical dilemmas such as physician assisted dying, disclosure of medical errors and relationship with pharmaceutical companies.

There have been few studies assessing knowledge, attitude and practice of healthcare ethics among doctors and nurses in resource poor countries such as ours. Moreover, there has been no such study prior to this in Nepal, specifically to assess the status of knowledge, attitude and practice of healthcare ethics among resident doctors and ward nurses in the same setting. Such studies would be important to monitor ethical practices and improve patient outcomes. Therefore, we hypothesized that there is paucity in knowledge, attitude and practice of health care ethics among health practitioners in Nepal.

## Methods

### Study settings

A cross-sectional study was performed among resident doctors and ward nurses of Tribhuvan University Teaching Hospital (TUTH) which is one of the biggest and reputed medical institutions in Nepal. The hospital employs 140 resident doctors enrolled in various postgraduate programmes and 250 ward nurses and around 0.4 million patients benefit from medical services of the hospital annually.

### Study population

In TUTH, PG (Post Graduate) trainee medical doctors are called as resident doctors who are often consulted first for all new patients and are responsible for supervision and management of patients in the wards. Similarly nurses in the ward, work in coordination and supervision to serve admitted patients. Status of knowledge, attitude and perception on health care ethics in TUTH is therefore best reflected from resident doctors and nurses.

### Questionnaire and variables

A 30 item questionnaire from Barbados and 34 item questionnaire from India were adapted [20, 21]. Out of 30 items from Barbados study 13 items were used and 13 items out of 34 item questionnaire from India were used. Remaining items from these questionnaires were not relevant to Nepalese setting. The selection of items from these questionnaires was made in order to make the questionnaire locally appropriate allowing incorporation of Nepal Medical Council Norms on Medical ethics adhering to the objective of this study [11].

The original questionnaire was in English. The questionnaire was translated to Nepali language and back translated to English by a bilingual translator to ensure consistency. The final questionnaire had 30 questions including 4 questions on socio-demographic characteristics (see Additional file 1). After the translation and back translation, it was pretested among 5 doctors and 5 nurses to assess the comprehensibility of questions. Any ambiguity in questions was corrected.

Ethnicity was classified into advantaged and disadvantaged according to Health Management Information System Classification of Nepal [22, 23]. Advantaged ethnic groups in general are privileged in terms of socio-economic status (education, economy, jobs and birthplace -urban versus rural).

We assessed participant knowledge about medical ethics codes namely Hippocrates codes, Nuremberg codes and Helsinki codes by asking key principles of them. Correct answer was marked “yes”,and insufficient details and lack of awareness about the codes was marked “no” to the knowledge of respective medical codes.

The second part of questionnaire consisted of 22 questions on different ethical issues ethical issues on which the respondents agreed or disagreed with the statements pertaining to adherence to patient will, confidentiality, autonomy, paternalism, abortion, physician-assisted dying, informed consent etc. The respondents were required to answer whether they agreed or disagreed with the statements presented. The final part of the questionnaire consisted of information depicting the source of knowledge for learning ethics and law as well as preference in consulting on a legal or ethical problem. On this final part of the questionnaire, multiple responses from the participants were allowed.

### Sampling procedure and data collection

A total of 135 resident doctors and 250 ward nurses were on the roster for the month of January, according to the records of hospital administration on 27th of January. All resident doctors were introduced about the study and asked for verbal consent, however, 5 were either at leave or refused to participate in the study. Similarly, every second nurse from the list in the roster was at first introduced about the study and asked for verbal consent. After obtaining the verbal agreement, self-administered questionnaire were distributed. However, 7 nurses were at leave and 3 refused to participate in the study. Total of 130 resident doctors and 120 ward nurses were provided questionnaire over the period of 15 days.

From total of 250 questionnaires, 210 were returned, out of which six questionnaires were incompletely filled and were excluded from the analysis. The study population consisted of 118 resident doctors and 86 ward nurses (*n* = 204, response rate was 84 %).

### Ethics

The study protocol was exempted from review by the Institutional Review Board of Institute of Medicine, Tribhuvan University, Nepal. All the resident doctors and nurses were asked for verbal consent before distributing the questionaries. Additionally, an informed consent form which described the study objectives was attached to the questionnaire, which the participants marked “yes or agree” if they wanted to proceed. Participation in the study was voluntary. No incentives were provided for participation.

### Data analysis

Data was analysed using Statistical Package for Social Sciences (SPSS) version 20.0 for windows. Data analysis was done using proportions and percentage. For the comparison of ethical attitudes among doctors and nurses, Chi Square test was employed. A Cramer’s V value was obtained to determine the strength of the difference in their opinions. The Cramer’s value of < 0.1, 0.1–0.5, >0.5 was used for small, medium and large respectively to measure the effect size. *P*-value < 0.05 was considered statistically significant.

## Results

### Demographic details

Out of the total of 204 respondents, 56.86 % (118) were resident doctors and the remaining participants (86) were ward nurses. The mean age for doctors was 28.66 years (SD = 1.89) and for nurses 27.69 years (SD = 6.97) (Table 1). The majority of the participants among doctors were males (67.8 %) whereas all nurses were females. Furthermore, most of the participants were from advantaged ethnic groups and from urban areas according to their place of birth.

**Table 1:** Demographic characteristics of the respondents

Characteristics	Job Category	
	Doctors (*N* = 118)	Nurses (*N* = 86)
Age(Mean ± SD)	28.66 ± 1.89 years	27.69 ± 6.97 years
Sex		
Male	80 (67.8)	-
Female	38 (32.2)	86 (100)
Ethnicity		
Advantaged	89 (75.4)	50 (58.1)
Disadvantaged	29 (24.6)	36 (41.9)
Place of Birth		
Urban	81 (68.6)	56 (65.1)
Rural	37 (31.4)	30 (34.9)

Data are frequencies (percentages)

### Source of knowledge on medical ethics

More than two thirds of the doctors preferred lectures on ethics, followed by books, as the instruments for learning ethics and law (Table 2). On the other hand, nearly 90 % of the nurses preferred journals and books on ethics as the instruments for learning ethics and law.

**Table 2:** Preferred instruments for learning about ethics and lawInstruments for learning ethics and law among doctors and nurses

Instruments for learning ethics and law	Doctors (*N* = 118)	Nurses (*N* = 86)
Ethics journals	69 (59.0)	74 (89.2)
Books on ethics	73 (62.4)	74 (89.2)
General texts	38 (32.5)	33 (39.8)
Media (Newspaper/TV)	49 (41.9)	55 (66.3)
Workshops	66 (56.4)	50 (60.2)
Lectures (UG/CME)	79 (67.5)	47 (56.6)
Panel discussions	60(51.3)	36 (43.4)
Case conferences	52 (44.4)	46 (55.4)

*UG* Undergraduate lectures, *CME* Continuing Medical Education lectures. Data are frequencies (percentage)

### Knowledge on ethical codes

Among the doctors, two thirds knew the content of Hippocratic Oath. Of the nurses, only a half knew the content of it. Similarly, about 90 % of doctors and nurses did not know the content of Nuremberg code and over 85 % of them did not know about the content of Helsinki Declaration (Table 3).

**Table 3:** Knowledge on ethical codes among doctors and nurses

Ethical codes	Job category		
	Doctors (*N* = 118)		Nurses (*N* = 86)
Knew the content of Hippocratic Oath	Yes	79 (66.9)	21 (49.0)
	No	39 (33.1)	65 (51.0)
Knew the content of Nuremberg Code	Yes	12 (10.2)	10 (10.8)
	No	106 (89.8)	76 (89.2)
Knew the content of Helsinki Code	Yes	17 (14.4)	7 (11.8)
	No	101 (85.6)	79 (88.2)

Data are frequencies (percentages)

### Preference for consultation

Tables 4 and 5 show the preference of the resident doctors and nurses for consultation regarding ethical and legal problem. Majority (71.2 %) of doctors preferred to consult their head of the department while most nurses (80.5 %) preferred to consult their supervisor on an ethical problem. Usually, the head of department in case of doctors and senior nurses in case of nurses are the supervisors.

**Table 4:** Preference in consulting on an ethical problem among doctors and nurses

Whom to consult	Doctors (*N* = 118)	Nurses (*N* = 86)
Colleague	64 (54.2)	47 (57.3)
Supervisor	64 (54.2)	66 (80.5)
Head of Department	84 (71.2)	46 (56.1)
Chief of Medical Staff	41 (34.7)	47 (57.3)
Matron	11 (9.3)	53 (64.6)
Hospital Administrator	44 (37.3)	47 (57.3)
Ethics Committee	54 (45.8)	61 (74.4)
Professional Association	35 (29.7)	39 (47.6)
Text,Internet	5 (4.2)	22 (26.8)
Close friend/family	12 (10.2)	30 (36.6)

Data are frequencies (percentages)

**Table 5:** Preference in consulting on a legal problem

Whom to consult	Doctors (*N* = 118)	Nurses (*N* = 86)
Colleague	57 (49.1)	35 (42.2)
Supervisor	70 (60.3)	67 (80.7)
Chief of Medical Staff	56 (48.3)	58 (69.9)
Matron	10 (8.6)	61 (73.5)
Hospital Administrator	72 (62.1)	58 (69.9)
Professional insurance company	11 (9.5)	19 (22.9)
Trade Union	5 (4.3)	17 (20.5)
Lawyer	78 (67.2)	59 (71.1)

Data are frequencies (percentages)

Similarly, the majority (67.2 %) of doctors preferred to consult a lawyer while majority of the nurses (80.7 %) preferred to consult their supervisor on a legal problem.

### Issues in different aspects of health care ethics

Table 6 shows the attitude towards different aspects of healthcare ethics among doctors and ward nurses. There was a statistically significant difference in attitude between resident doctors and ward nurses with respect to adherence to patient’ wishes (66.9 % doctors agreed versus 80.2 % nurses agreed, *p* = 0.036), informing close relative about patient’s opinion (77.1 % doctors agreed versus 96.5 % nurses agreed, *p* = <0.001), seeking consent for treating children (72.0 % doctors agreed versus 86.0 % nurses agreed, *p* = 0.017),conducting abortion if law allowed (45.7 % doctors agreed versus 69.7 % nurses agreed, *p* < 0.001), paternalistic attitude of doctors while on disagreement between patients/families and health professionals (20.3 % doctors agreed versus 9.3 % nurses agreed, *p* = 0.032), adherence to confidentiality (2.5 % doctors agreed versus 11.6 % nurses agreed, *p* = 0.009), documentation of neurological examination and blood pressure without being done (9.3 % doctors agreed versus 23.3 % nurses agreed, *p* = 0.006), necessity of incorporating medical ethics in undergraduate curriculum (97.4 % doctors agreed versus 81.3 % nurses agreed, (*p* < 0.001) and refusal to examine a female patient in the absence of a chaperone (33.9 % doctors agreed versus 52.3 % nurses agreed, *p* = 0.008) (Table 6). More nurses agreed to above nine questions compared to doctors except for necessity of incorporating medical ethics in undergraduate curricula and paternalistic attitude of doctor while on disagreement.

**Table 6:** Issues in different aspects of healthcare ethics

Issues in healthcare ethics	Agree	Disagree	Chi square	Cramer’s V	*P*-value
Patient wishes must always be adhered to					
Doctors	79 (66.9)	39(33.1)	4.40	0.14	0.036^*^
Nurses	69(80.2)	17(19.8)			
Patient should always be informed of wrong doing					
Doctors	83(70.3)	35(29.7)	0.008	0.006	0.930
Nurses	60(69.7)	26(30.3)			
Confidentiality is not important					
Doctors	9 (7.6)	109 (92.4)	1.53	0.87	0.215
Nurses	3 (3.5)	83 (96.5)			
Doctor should do irrespective of patient’s opinion					
Doctors	71(60.2)	47(39.8)	0.90	0.06	0.341
Nurses	46(53.5)	40(46.5)			
Close relative should always be told about patient’s opinion					
Doctors	91(77.1)	27(22.9)	14.91	0.27	<0.001*
Nurses	83(96.5)	3(3.5)			
Children should never be treated without the consent of their parents					
Doctors	85(72.0)	33(28.0)	5.58	0.16	0.017*
Nurses	74(86.0)	12(14.0)			
If law allows abortion, doctors cannot refuse to do abortion					
Doctors	54(45.7)	64(54.3)	11.62	0.23	0.001*
Nurses	60(69.7)	26(30.3)			
If there is disagreement between patients/families and health care professionals about treatment decisions, doctors decision should be final					
Doctors	24(20.3)	94(79.7)	4.58	0.15	0.032*
Nurses	8(9.3)	78(90.7)			
Ethical conduct is only important to avoid legal action					
Doctors	13(11.0)	105(89.0)	2.34	0.10	0.125
Nurses	16(18.6)	70(81.4)			
Ethics as part of a syllabus should be taught in every medical/nursing teaching institution					
Doctors	116(98.3)	2(1.7)	0.097	0.02	0.755
Nurses	85(98.8)	1(1.2)			
It is very difficult to keep confidentiality, so it should be abandoned					
Doctors	3(2.5)	115(97.5)	6.88	0.18	0.009*
Nurses	10(11.6)	76(88.4)			
Doctors are receiving income from referring patients for medical tests					
Doctors	80(67.8)	38(32.2)	1.17	0.076	0.874
Nurses	52(60.5)	34(39.5)			
Consent is only required for surgeries, not for tests and medicines					
Doctors	15(12.7)	103(87.3)	0.24	0.03	0.623
Nurses	9(10.5)	77(89.5)			
Copying answers in degree examinations is bad/sin					
Doctors	101(85.5)	17(14.5)	0.11	0.023	0.740
Nurses	75(87.2)	11(12.8)			
Acceptability in recording nervous system examination or blood pressure as normal when it has not been done for completion of documentation					
Doctors	11(9.3)	107(90.7)	7.49	0.19	0.006*
Nurses	20(23.3)	66(76.7)			
If a patient wishes to die, he or she should be assisted in doing so					
Doctors	11(9.3)	107(90.7)	1.06	0.07	0.302
Nurses	12(14.0)	74(86.0)			
Doctors are influenced by drug company inducements, including gifts					
Doctors	95(80.5)	23(19.5)	0.02	0.01	0.874
Nurses	70(81.4)	16(18.6)			
In order to prevent transmission of tuberculosis, disclosure of tuberculosis positive status to neighbours should be done					
Doctors	62(52.5)	56(47.5)	1.03	0.07	0.310
Nurses	39(45.3)	47(54.7)			
It is ethical to refuse a patient given a situation, a male doctor needs to examine a female patient and female attendant is not available					
Doctors	40(33.9)	78(66.1)	6.95	0.18	0.008*
Nurses	45(52.3)	41(47.7)			
Presence of interest in learning healthcare ethics					
Doctors	114(96.6)	4(2.0)	2.97	0.12	0.085
Nurses	86(100.0)	0(0.0)			
Doctors/nurses must serve hard to reach remote areas and under-served population					
Doctors	105(89.0)	13(11.0)	0.752	0.061	0.386
Nurses	73(84.9)	13(15.1)			
Necessity of incorporating medical ethics in undergraduate curriculum					
Doctors	115(97.4)	3(2.6)	15.19	0.27	<0.001*
Nurses	70(81.3)	16(18.7)			

Data are frequencies (percentages), *p < 0.05 is statistically significant

The attitudes of the doctors however, were not substantially different from those of the nurses in a number of responses showing either a small or medium Cramer’s value (<0.5). The doctors were not significantly different from nurses in a number of aspects, such as informing patient of wrongdoing (*p* = 0.930), importance of confidentiality (*p* = 0.215), paternalism (doing irrespective of patient’s opinion) (*p* = 0.341), teaching ethics as a part of the syllabus (*p* = 0.755), importance of ethical conduct (*p* = 0.125), benefit for doctors for referring medical tests (*p* = 0.874), consent for tests and medicines (*p* = 0.623), cheating in degree exams (*p* = 0.740), physician-assisted dying (*p* = 0.302), luring of doctors by drug companies (*p* = 0.874), disclosure of tuberculosis status (*p* = 0.310), interest in learning healthcare ethics (*p* = 0.085), opinion on serving in remote areas and for underserved populations (*p* = 0.386).

Nearly all resident doctors (96.6 %) and all ward nurses (100 %) reported an interest in learning healthcare ethics. Similarly,the majority of them supported the incorporation of medical ethics in undergraduate curriculum and its teaching in every medical/nursing institution. About 80 % of resident doctors and ward nurses believed that doctors are influenced by drug company inducements. Furthermore, nearly 90 % of them leaned against physician-assisted death.

## Discussion

Our study showed significant knowledge on healthcare ethics among doctors and nurses; however, knowledge on the central ethical codes in clinical practice was poor, particularly, Nuremberg Code and Helsinki Declaration were not familiar amongst respondents. Studies from India, where the modality of imparting medical education is somewhat similar to Nepal, also revealed that the majority of respondents were unaware of Nuremberg code and Helsinki Declaration [24, 25]. Encouragingly, both doctors and nurses seemed to express interest in learning healthcare ethics. A substantial proportion of them respondents felt that incorporating ethics as a part of the syllabus in the curricula is necessary and should be taught in every medical school and nursing institution. This warrants the urgent prioritization for incorporation of medical ethics in curricula.

In our study, doctors preferred to learn ethics and law with lectures, books and journals while nurses preferred ethics journals and books. Consistent with our findings, previous studies have pointed lectures and books as predominant sources of learning ethics among medical students, doctors and nurses [26–28].

In addition, several studies have shown that learning ethics through presentation of real cases, training and during work were predominant sources of learning healthcare ethics [20, 27, 29]. This further implies that apart from teaching health care ethics through lectures, training at workplace and subscription of journals on ethics and books can be highly promising.

A substantial percentage of resident doctors and nurses were unaware of the content of Hippocratic Oath. Similarly, the majority of them did not know about the content of Nuremberg code and Helsinki Declaration. These findings explicitly illustrated the dismal knowledge on the most basic ethical principles and research ethics among health professionals. The findings on poor knowledge on ethical codes are in line with previous studies conducted both in high and low income countries [20, 27].

The majority of the resident doctors preferred to consult their head of department while the nurses preferred to consult their supervisor on an ethical problem. In general, expectedly, doctors and nurses consult their seniors from the workplace. In our study, surprisingly, very few of them opted to consult their close friend. This implies that when the doctors and nurses encounter an ethical challenge, they tend to settle it at the department or through consultation with the supervisors. Additionally, it could be that doctors and nurses perceive that supervisors at workplace could solve the ethical challenges efficiently. .

On legal issues, doctors opted to consult a lawyer and nurses opted to consult their supervisor. Neither affiliation to any trade union nor registration in a professional insurance company was available for resident doctors and nurses in Tribhuvan University Teaching Hospital; therefore, consultation to lawyer must have been an obvious option.

Our study elucidates that doctors and nurses differ in their attitudes pertaining to practical ethical issues such as informing close relative, consent for treating children, abortion, paternalistic attitude while on disagreement, adherence to confidentiality, refusal to examine a female patient in the absence of female attendant and necessity of incorporation of medical ethics as a part of syllabus. Monsudi et al. and Imran et al. argued that different intensity of professional training creates the difference in opinions among health staffs [30, 31]. Medical graduates receive hardly ten hours of lectures on medical ethics during their 5 years of medical degree, similarly, nurses receive limited orientation during their 3 years of training in Nepal. Therefore, this discrepancy of opinions in our study population of doctors and nurses could be attributed to personalized judgment in the absence of knowledge on ethics. This implies that the uniformity in health care ethics is urgently required regardless of various professions within the health care that includes doctors, nurses, paramedics and lab staffs by imparting knowledge on medical ethics which should be similar across the profession. This further demands the discussion and comparison of knowledge, attitude and practices in health care ethics within the profession, institution and disciplines and additionally comparison amongst them.

In this study, majority of doctors and nurses leaned against physician-assisted dying. In Nepal, state jurisdiction has not legalized it. Physician-assisted suicide and euthanasia are widely debated. Physician-assisted dying is viewed in the facets of contradicting a physician’s role, devaluing human life and inaccessibility for the poor [32, 33]. The study results showed that Nepalese doctors and nurses are committed to their professional responsibilities and they cannot do what the state jurisdiction does not allow. Contrarily, physicians in countries and regions where physician-assisted dying is legal might allow this practice, however, the rationale and legality of physician assisted dying has been highly debatable amongst physician in these countries.

Considerably, resident doctors and nurses in this study are convinced that doctors are influenced by inducements from pharmaceutical companies. In Nepal, doctors are lured by offers of drug companies in the form of gifts, sponsorship of seminars and symposiums or continuing medical education in return for prescribing the medications of their brand [34]. This compels poly-pharmacy, creates biases on prescription and increases economic burden in care seekers [35, 36].

This study is the first to explore the knowledge, attitude and practice of medical ethics in Nepal, one of the few studies conducted in South Asia. The survey instrument in this study was used elsewhere and was adapted to comply with the objective of the study and to make it locally appropriate.

### Limitation

In this study, sampling of doctors and ward nurses was confined to a single tertiary hospital. The reported responses on medical ethics might have suffered from social desirability bias, as participants might have responded what is viewed favourably by others ,not what they perceived ‘ethical’ to responded questions. Our assessment of knowledge on medical ethics was limited to knowledge on three codes of medical ethics. Further assessment of knowledge would have been possible by inclusion of knowledge on principles of biomedical ethics namely ‘Autonomy’, ‘Beneficence’, ‘Maleficence’ and ‘Justice’. However, inclusion of these principles into the questionnaire would need a pilot study with further work on instrumentation of questionnaire for analysis.

## Conclusions

Medical ethics is one of the much neglected topics in healthcare in resource poor settings. This study demonstrated that significant proportion of doctors and nurses were unaware of the universally recognized ethical principles (three major documents on medical ethics) which are essential part of clinical practice. A majority of respondents in this study showed interest in learning medical ethics and recommended for inclusion of medical ethics in the curriculum. Provision of information on medical ethics in medical institution through subscription of journals and books on ethics in addition to the lectures and training on medical ethics at workplace can significantly raise the current paucity of knowledge in health care workers.

## Additional file

Additional file 1: Questionnaire. (DOCX 30 kb)

## Abbreviation

TUTH: Tribhuvan University Teaching Hospital
