# Supplementary material for: Knowledge, attitude and practice of healthcare ethics among resident doctors and ward nurses from a resource poor setting, Nepal
Source: BMC Med Ethics. 2016 Nov 8;17:68. doi: 10.1186/s12910-016-0154-9 (PMC5100232; doi:10.1186/s12910-016-0154-9)
Supplement: Additional file 1: — Questionnaire. (DOCX 30 kb) [file 12910_2016_154_MOESM1_ESM.docx]

**Knowledge, Attitude and Practice towards Healthcare Ethics among resident doctors and ward nurses in Tribhuvan University Teaching Hospital, Nepal**

Hello, I am Samaj Adhikari, an intern doctor in Tribhuvan University Teaching Hospital (TUTH), Institute of Medicine. I and my colleagues are conducting a research study on knowledge, attitude and practice towards health care ethics among doctors and nurses in TUTH. This questionnaire will take approximately 20 minutes to complete. Your participation in this study is completely voluntary, and all your responses will be kept confidential. We hope you will support this study by providing valuable information.

Do you agree to participate? Yes No

ID:

**Please mark (√ ) on the appropriate answer in the questionnaire below.**

| **S.N.** | **Demographic characteristics** |  |  |
| --- | --- | --- | --- |
|  | Age | ……………………. |  |
|  | Sex | ……………............. |  |
|  | Ethnicity |  |  |
|  | Place of birth | **Urban** | **Rural** |
|  | **Issues in healthcare ethics** | **Agree** | **Disagree** |
|  | Patients wishes must always be adhered to |  |  |
|  | Patient should always be informed of wrong doing by anyone involved in his/her treatment. |  |  |
|  | Confidentiality is not so important aspect of treatment. |  |  |
|  | Doctors should do their best for the patient irrespective of the patient’s opinion. |  |  |
|  | Close relatives should be told about patient’s condition. |  |  |
|  | Children should not be treated without consent of their parents. |  |  |
|  | If law allows abortion, doctors cannot refuse to do abortion |  |  |
|  | If there is Disagreement between patients/families and health care professionals about treatment decisions, doctors decision should be final. |  |  |
|  | **Issues in practice of healthcare ethics** | **Agree** | **Disagree** |
|  | Ethical conduct is only important to avoid legal action. |  |  |
|  | Ethics as a part of syllabus should be taught in every medical/nursing teaching institution. |  |  |
|  | It is very difficult to keep confidentiality, so it should be abandoned. |  |  |
|  | In your opinion do you think that doctors are receiving income from referring patients for medical tests? |  |  |
|  | Consent is required only for surgeries, not for tests and medicines. |  |  |
|  | Copying answers in degree examinations is bad/sin. |  |  |
|  | Writing Nervous system examination- normal or blood pressure normal when it hasn’t been done is acceptable because it is important for documentation. |  |  |
|  | If a patient wishes to die, he or she should be assisted in doing so no matter what their illness. |  |  |
|  | In your opinion do you think that doctors are influenced by drug company inducements, including gifts? |  |  |
|  | In order to prevent transmission of TB, disclosure of TB positive status to neighbors should be done. |  |  |
|  | Given a situation, a male doctor need to examine a female patient & female attendant is not available; in your opinion is it ethical to refuse the patient? |  |  |
|  | Do you have interest in learning healthcare ethics? |  |  |
|  | Do you think doctors/nurses must serve hard to reach areas and underserved population? |  |  |
|  | Do you think it is necessary to incorporate medical ethics in undergraduate curriculum? |  |  |

**Please mark (√ ) on the appropriate answer in the questionnaire below.**

***Multiple Responses (multiple responses are acceptable)***

|  | **Instruments for learning ethics and law** |  |
| --- | --- | --- |
|  | Ethics journals |  |
|  | Books on ethics |  |
|  | General texts |  |
|  | Media (Newspapers/TV) |  |
|  | Workshops |  |
|  | Lectures (UG/CME) |  |
|  | Panel discussions |  |
|  | Case conferences |  |
|  | **Preference in consulting on a legal problem** |  |
|  | Colleague |  |
|  | Supervisor |  |
|  | Chief of Medical staff |  |
|  | Matron |  |
|  | Hospital Administrator |  |
|  | Professional insurance company |  |
|  | Trade Union |  |
|  | Lawyer |  |
|  | **Preference in consulting on an ethical problem** |  |
|  | Colleague |  |
|  | Supervisor |  |
|  | Head of Department |  |
|  | Chief of Medical staff |  |
|  | Matron |  |
|  | Hospital Administrator |  |
|  | Ethics Committee |  |
|  | Professional Association |  |
|  | Text, Internet |  |
|  | Close friend/family |  |
|  | **Knowledge about Ethical Codes** |  |
|  | Do you know the content of Hippocratic oath? Please describe in the box below. |  |
|  |  |  |
|  |  |  |
|  | Do you know the content Nuremberg Code? Please describe in the box below. |  |
|  |  |  |
|  |  |  |
|  | Do you know the content Helsinki Declaration? Please describe in the box below. |  |
|  |  |  |
|  |  |  |

Thank you for your support
